# New Details of HCV NS3/4A Proteinase Functionality Revealed by a High-Throughput Cleavage Assay

**DOI:** 10.1371/journal.pone.0035759

**Published:** 2012-04-27

**Authors:** Sergey A. Shiryaev, Elliot R. Thomsen, Piotr Cieplak, Eugene Chudin, Anton V. Cheltsov, Mark S. Chee, Igor A. Kozlov, Alex Y. Strongin

**Affiliations:** 1 Inflammatory and Infectious Disease Center, Sanford-Burnham Medical Research Institute, La Jolla, California, United States of America; 2 R&D Department, Prognosys Biosciences Inc., La Jolla, California, United States of America; University of Pittsburgh, United States of America

## Abstract

**Background:**

The hepatitis C virus (HCV) genome encodes a long polyprotein, which is processed by host cell and viral proteases to the individual structural and non-structural (NS) proteins. HCV NS3/4A serine proteinase (NS3/4A) is a non-covalent heterodimer of the N-terminal, ∼180-residue portion of the 631-residue NS3 protein with the NS4A co-factor. NS3/4A cleaves the polyprotein sequence at four specific regions. NS3/4A is essential for viral replication and has been considered an attractive drug target.

**Methodology/Principal Findings:**

Using a novel multiplex cleavage assay and over 2,660 peptide sequences derived from the polyprotein and from introducing mutations into the known NS3/4A cleavage sites, we obtained the first detailed fingerprint of NS3/4A cleavage preferences. Our data identified structural requirements illuminating the importance of both the short-range (P1–P1′) and long-range (P6-P5) interactions in defining the NS3/4A substrate cleavage specificity. A newly observed feature of NS3/4A was a high frequency of either Asp or Glu at both P5 and P6 positions in a subset of the most efficient NS3/4A substrates. In turn, aberrations of this negatively charged sequence such as an insertion of a positively charged or hydrophobic residue between the negatively charged residues resulted in inefficient substrates. Because NS5B misincorporates bases at a high rate, HCV constantly mutates as it replicates. Our analysis revealed that mutations do not interfere with polyprotein processing in over 5,000 HCV isolates indicating a pivotal role of NS3/4A proteolysis in the virus life cycle.

**Conclusions/Significance:**

Our multiplex assay technology in light of the growing appreciation of the role of proteolytic processes in human health and disease will likely have widespread applications in the proteolysis research field and provide new therapeutic opportunities.

## Introduction

Hepatitis C is a viral disease with over 180 million people infected worldwide. The virus primarily affects the liver and 80% of infected patients develop chronic hepatitis. The HCV genome is a single-stranded, 9600 nucleotide long RNA molecule of positive polarity. This RNA has a long open-reading frame that is flanked at both ends by short non-translated regions. Protein synthesis is mediated by an internal ribosome-entry site (IRES) http://www.nature.com/nrd/journal/v1/n11/full/nrd942.html – B5that binds directly to ribosomes [1]. After infection of the host cell, the liberated viral RNA is translated into a single polyprotein that consists of three structural proteins (Core, E1 and E2) and seven non-structural (NS) proteins arranged in the order NH_2_-C-E1-E2-p7-NS2-NS3-NS4A-NS4B-NS5A-NS5B-COOH. The precursor is then proteolytically cleaved into ten individual proteins by viral and cellular proteinases [2], [3]. The structural proteins are used to assemble new virus particles. The NS proteins participate in the replication of the viral genome [4]. This replication is catalyzed by the ATP-dependent NS3 helicase, which unwinds double-stranded RNA into single strands, and the NS5B RNA-dependent RNA polymerase [5]. In the course of RNA replication, the viral genome acts as a template for the synthesis of negative-strand RNA, which then acts as a template for positive-strand RNA [6], [7].

There are six genotypes (1 through 6) of HCV, which are unequally distributed in different parts of the world [8]. Genotype 1 is the most common HCV genotype in the US and Europe. Approximately 80% of HCV infections in the US are of genotype 1. Because NS5B, the RNA-dependent RNA polymerase, misincorporates bases at a high rate, HCV constantly mutates as it replicates [9]. The process of constant mutation leads to multiple quasi-species of HCV and helps the virus to evade both the host immune response and anti-virals. These multiple mutations modify the polyprotein sequence and, as a result, make the virus resistant to inhibitors [10].

**Figure 1:**
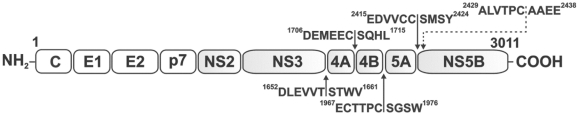
HCV polyprotein (**genotype 1a; GenBank accession P26664**) **with the NS3/4A cleavage sites.** Non-structural (NS) proteins are shaded gray. Conventional NS3/4A cleavage sites in the NS3-NS4A, NS4A-NS4B, NS4B-NS5A and NS5A-NS5B junction regions are shown by solid arrows. The putative cleavage site (^2429^ALVTPC-AAEE^2438^) in the NS5A-NS5B junction is shown by a dashed arrow. The C–E1, E1–E2, E2–p7 and p7-NS2 junctions are cleaved by host cell signal peptidase. The NS2-NS3 junction is cleaved by NS2/3 auto-proteinase. C, core protein.

The NS3 proteinase catalytic domain represents the N-terminal, ∼180-residue, portion of the 631-residue NS3 protein. The C-terminal domain of NS3 encodes the ATP-dependent RNA helicase. The NS3 catalytic domain alone is inactive and requires either the full-length NS4A co-factor or, at least, its 14-residue hydrophilic central portion for cleavage activity *in vitro* and *in vivo* [11]–[13]. NS4A is a 54 residue protein, with a hydrophobic N-terminus and a hydrophilic C-terminus. Following binding with NS4A, the NS3 domain is re-arranged leading to the proper alignment of His-57, Asp-81, and Ser-139 of the catalytic triad [14], [15]. NS3/4A exhibits a Zn-binding site that comprises Cys-97, Cys-99, Cys-145 and His-149, and that serves a structural role [16]. The two-component HCV NS3 serine proteinase (NS3/4A) is responsible for proteolytic processing of the viral polyprotein at the NS3-NS4A, NS4A-NS4B, NS4B-NS5A and NS5A-NS5B junctions (Fig 1) [17], [18]. The functional importance of NS3/4A makes it a prime anti-viral drug target [19], [20].

Current understanding of the cleavage preferences of NS3/4A is based both on a few cleavage site sequences in the HCV polyprotein and on the analysis of a limited set of synthetic peptides [21]–[27]. Evidence suggests that NS3/4A exhibits narrow cleavage specificity and a strong preference for Cys and Ser at the P1 and P1′ residue positions, respectively. Relative values of other residues that are proximal to the substrate scissile bond are not well established.

To elucidate the cleavage preferences of NS3/4A in depth and to shed additional light on HCV polyprotein processing, we employed a new approach for the multiplexed analysis of protease activity. Analysis of these more extensive cleavage results clarified the interactions of NS3/4A with its cleavage targets and provided further evidence of the indispensable role of this proteinase in the polyprotein processing and viral life cycle.

## Results

### Multiplexed cleavage assay

We utilized a new approach for the scalable multiplexed analysis of protease activity (Kozlov IA, Thomsen ER, Munchel SE, Villegas P, Capek P^,^ Gower AJ, Pond P, Chudin E and Chee MS. A Highly Scalable Peptide-Based Assay System for Proteomics, submitted). This novel profiling methodology employs cDNA-peptide fusions and has been validated using thousands of peptide sequences representing substrates for several proteinases, including furin, enterokinase, thrombin, caspases, NS2B-NS3 proteinases of Dengue and West Nile viruses, and NS3/4A. This methodology greatly accelerates determining the cleavage efficiency of the peptide substrates leading to the in-depth understanding of proteinase cleavage preferences.

For this project, the synthesized peptides contained constant N-terminal and C-terminal sequences (Cys-Ala and Ala-Gly-Asn-Ala-Ser-Ala-Ser-Ala, respectively) flanking an 8-residue sequence derived from the HCV polyprotein. When specifying the peptide sequences below, we normally omit the constant regions. Therefore, statements that refer to 8-residue peptides are related to the variable, HCV-specific portion of a longer peptide that is conjugated to a cDNA oligonucleotide.

cDNA-peptide fusions were immobilized on magnetic beads using an affinity tag attached to the N-terminus of all peptides. Following proteolytic cleavage of the peptide portion of the fusion, the corresponding cDNA template was released in solution. The cleaved peptide substrates were then identified by sequencing the released cDNA portion of the fusion. We used next-generation sequencing to enable the high throughput readout of our screening assay.

### Global analysis of the NS3/4A cleavage preferences

To focus more specifically on the cleavage preferences of NS3/4A and to span the entire sequence of the 3,011 residue HCV1 polyprotein precursor (GeneBank Accession P26664) we used a two-residue protein walking approach. The sequence of the first 8-mer peptide started from the N-terminus of the polyprotein precursor. There was a two residue offset resulting in a 6 residue overlap of the upstream and downstream peptides. Hence, each residue position of the polyprotein was included in the sequence of the multiple distinct peptides. As a result of this walking, the effects of each of the P6–P4′ residue positions on the NS3/4A cleavage efficiency could be ascertained. The set of 1,503 peptide conjugates (or 1,663 including all of the control peptides), tiled in this fashion, fully covered the polyprotein sequence.

We also synthesized over 800 mutant peptides, the sequence of which overlapped either with the sequence of the NS3-NS4A (^1652^DLEVVT↓STWV^1661^), NS4A-NS4B (^1706^DEMEEC↓SQHL^1715^), NS4B-NS5A (^1967^ECTTPC↓SGSW^1976^) and NS5A-NS5B (^2415^EDVVCC↓SMSY^2424^) junctions or with those of the potential host cell targets of NS3/4A, interferon-β promoter stimulator protein 1 (IPS-1) (160 peptides) and Toll/IL1R (TIR) domain-containing adaptor molecule (TRIF; TCAM1) (120 peptides) (Supplemental Tables S1, S2, and S3; Tables 1 and 2). These peptide conjugates were also treated with NS3/4A *in vitro* and their cleavage efficiency was measured in parallel with the 1,503 original peptide conjugates. The combined data were analyzed to generate a comprehensive representation of the cleavage preferences of NS3/4A. The selected peptides were also co-incubated with several additional proteinases including furin and thrombin. The resistance of these peptides to these two proteinases confirms the selectivity and accuracy of our cleavage technology.

**Table 1:** Protein walking: cleavage islands in the HCV polyprotein.

Peptides													Z-score		
NS3-NS4A junction													NS3/4A	TR	Furin
IMTCMSAD													–	–	–
	TCMSADLE												–	–	–
		MSADLEVV											–	–	–
			ADLEVVTS										–	–	–
				DLEVVTST									–	–	–
					LEVVTSTW								–	–	–
						***EVVTSTWV***							–	–	–
							VVTSTWVL						–	–	–
								TSTWVLVG					–	–	–
									TWVLVGGV				–	–	–
										VLVGGVLA			–	–	–
											VGGVLAAL		–	–	–
												GVLAALAA	–	–	–

NS4A-NS4B junction															
EVLYREFD													–	–	–
	LYREFDEM												–	–	–
		REFDEMEE											–	–	–
			FDEMEECS										8.8	–	–
				DEMEECSQ									7.0	–	–
					EMEECSQH								6.4	–	–
						***MEECSQHL***							6.6	–	–
							EECSQHLP						3.7	–	–
								CSQHLPYI					–	–	–
									QHLPYIEQ				–	–	–
										LPYIEQGM			–	–	–
											YIEQGMML		–	–	–
												EQGMMLAE	–	–	–

NS4B-NS5A junction															
LRRLHQWI													–	–	–
	RLHQWISS												–	–	–
		HQWISSEC											3.7	–	–
			WISSECTT										–	–	–
				SSECTTPC									5.8	–	–
					ECTTPCSG								4.3	–	–
						***TTPCSGSW***							4.3	–	–
							PCSGSWLR						–	6.9	–
								SGSWLRDI					–	–	–
									SWLRDIWD				–	–	–
										LRDIWDWI			–	–	–
											DIWDWICE		–	–	–
												WDWICEVL	–	–	–

NS5A-NS5B junction																		
WSTVSSEA																–	–	–
	TVSSEANA															–	–	–
		SSEANAED														–	–	–
			EANAEDVV													–	–	–
				NAEDVVCC												9.3	–	–
					EDVVCCSM											9.1	–	–
						***VVCCSMSY***										8.9	–	–
							CCSMSYSW									–	–	–
								SMSYSWTG								–	–	–
									SYSWTGAL							–	–	–
										SWTGALVT						–	–	–
											TGALVTPC*					4.6	–	–
												ALVTPCAA*				3.4	–	–
													VTPCAAEE*			3.9	–	–
														PCAAEEQK		–	–	–
															AAEEQKLP	–	–	–

Over 2660 peptides were cleaved by NS3/4A and by thrombin and furin (controls). The cleavage data of the peptide sequences (P4–P4′) which either overlap or are proximal to the NS3-NS4A (^1654^EVVT↓STWV^1661^), NS4A-NS4B (^1708^MEEC↓SQHL^1715^), NS5A↓NS5B (^1969^TTPC↓SGSW^1976^) and NS5A↓NS5B (^2417^VVCC↓SMSY^2424^) junction regions are shown here. Peptides which directly correspond to the known cleavage sites are both in bold and italicized. The asterisk marks the peptides overlapping a putative additional NS3/4A cleavage site (^2431^VTPC↓AAEE^2438^). Only the Z-score equal or above 3 are shown. Minus indicates Z-scores below 3. The peptide PCSGSWLR↓ was cleaved by a thrombin (TR) control while all of other peptides were resistant to both thrombin and furin controls. In addition, our set included multiple control cleavage peptides for furin, such as RKKR↓STSA (Z-score  = 6.0) and RRKR↓YAIQ (Z-score  = 7.2). These peptides were not cleaved by thrombin and NS3/4A. Our set also included multiple control cleavage peptides for thrombin such as VVPR↓GVNL (Z-score  = 10) and IEPR↓SFSQ (Z-score  = 13) which were not cleaved by furin and NS3/4A.

**Table 2:** Alanine scanning mutagenesis of the NS3/4A cleavage sequences.

Positions	NS3-NS4A ^1652^DLEVVT↓STWV^1661^				NS4A-NS4B^1706^DEMEEC↓SQHL^1715^				NS4B-NS5A^1967^ECTTPC↓SGSW^1976^				NS5A-NS5B^2415^EDVVCC↓SMSY^2424^			
WT, P6-P2′	DLEVVTST			-	DEMEECSQ			7.0	ECTTPCSG			4.3	EDVVCCSM			9.1
P6	***A***LEVVTST			-	***A***EMEECSQ			5.4	***A***CTTPCSG			-	***A***DVVCCSM			8.3
P5	D***A***EVVTST			-	D***A***MEECSQ			5.7	E***A***TTPCSG			-	E***A***VVCCSM			9.1
WT, P4-P4′		EVVTSTWV		-		MEECSQHL		6.6		TTPCSGSW		4.3		VVCCSMSY		8.9
P4		***A***VVTSTWV		-		***A***EECSQHL		4.1		***A***TPCSGSW		5.2		***A***VCCSMSY		6.5
P3		E***A***VTSTWV		-		M***A***ECSQHL		3.2		T***A***PCSGSW		3.0		V***A***CCSMSY		4.8
P2		EV***A***TSTWV		-		ME***A***CSQHL		5.6		TT***A***CSGSW		-		VV***A***CSMSY		8.8
P1		EVV***A***STWV		-		MEE***A***SQHL		-		TTP***A***SGSW		-		VVC***A***SMSY		4.0
P1'		EVVT***A***TWV		-		MEEC***A***QHL		6.7		TTPC***A***GSW		5.0		VVCC***A***MSY		8.3
P2'		EVVTS***A***WV		-		MEECS***A***HL		6.9		TTPCS***A***SW		4.5		VVCCS***A***SY		8.4
P3'		EVVTST***A***V		-		MEECSQ***A***L		7.4		TTPCSG***A***W		5.5		VVCCSM***A***Y		8.2
P4'		EVVTSTW***A***		-		MEECSQH***A***		5.1		TTPCSGS***A***		3.0		VVCCSMS***A***		8.3

Alanine scanning mutagenesis was used to systematically substitute amino acid residues at the P6–P4′ positions of the NS3-NS4A (^1652^DLEVVT↓STWV^1661^), NS4A-NS4B (^1706^DEMEEC↓SQHL^1715^), NS4B-NS5A (^1967^ECTTPC↓SGSW^1976^) and NS5A-NS5B (^2415^EDVVCC↓SMSY^2424^) junctions. Peptides were then cleaved NS3/4A. Two series of the 8-residue peptides were tested: the peptides which corresponded to the P6–P2′ and to the P4–P4′ positions. Only Z-scores above 3 are shown. Minus indicates Z-scores below 3.

**Figure 2:**
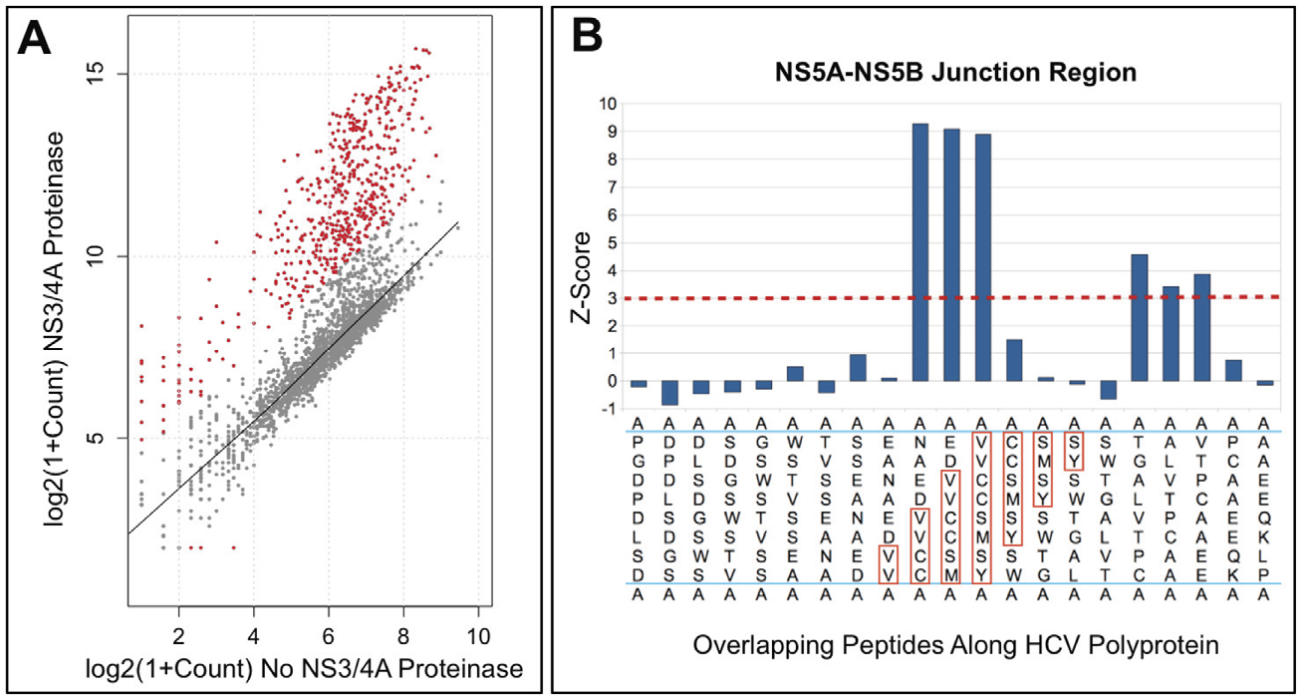
Results obtained after treatment of the 2,660 peptide substrate pool using NS3/4A. (**A**) A scatter plot of peptide abundances in which Y-axis and X-axis represent the peptides following NS3/4A cleavage and untreated controls, respectively. The peptide abundances were determined by sequencing counts of cDNAs corresponding to these peptides. The red points represent peptides that exhibited a statistically significant change in abundance because of NS3/4A proteolysis. Z-score of 3 (p<0.0014) was set as a cutoff, which corresponds to a false discovery rate below 0.01. (**B**) A set of the 21 overlapping 8-mer peptides derived from the NS5A-NS5B junction region sequence (^2415^EDVVCC↓SMSY^2424^). The Y-axis represents the Z-score for each peptide. The dotted red line represents Z-score = 3. Because the peptide sequences overlap, several adjacent peptides may contain sufficient recognition sequence to be cleaved. Peptide sequences are written vertically. Residue positions of the known NS3/4A cleavage site in the NS5A-NS5B junction region are highlighted (red boxes). The letter “A” at the start and at the end of each peptide sequence represents Ala residue from the flanking common regions at the N- and C-termini.

Specifically, the synthesized HCV peptides were incubated for 7.5, 15, and 240 min in the presence of purified NS3/4A at a 1∶10 enzyme-substrate molar ratio and processed as described in an accompanying paper by Kozlov IA, Thomsen ER, Munchel SE, Villegas P, Capek P^,^ Gower AJ, Pond P, Chudin E and Chee MS. A Highly Scalable Peptide-Based Assay System for Proteomics, submitted). The digest reactions were processed using our novel cleavage array technology. The cleavage signals were expressed as Z-scores. Under the hypothesis of no cleavage, z–scores have a normal distribution with a mean value that equals to 0 and a standard deviation of 1. We chose to reject the hypothesis of no cleavage for peptides with a Z-score greater than 3, which corresponds to p-value <1.35e^−3^. The recorded cleavage data are presented in Supplemental Table S1. The data obtained after cleavage of our 2,600 peptide set by NS3/4A are shown in Fig 2.

**Figure 3:**
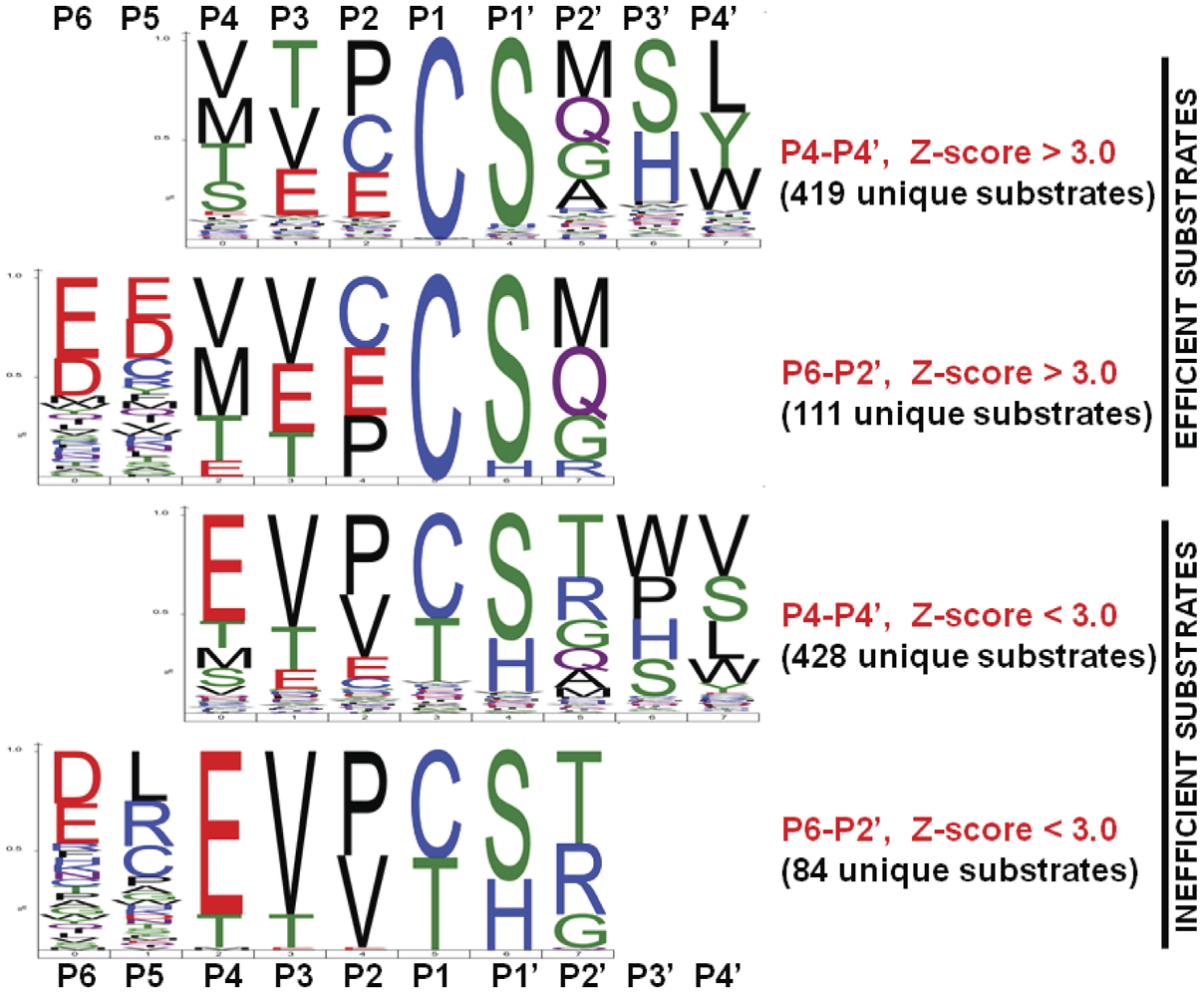
Frequency plot of the cleavage sequences of NS3/4A in a Weblogo format. The size of the symbol indicates the frequency of the individual residue occurrence at individual substrate positions relative to the P1–P1′ scissile bond. The frequencies were calculated using over 2,660 peptide sequences derived from the polyprotein and from introducing mutations to the known NS3/4A cleavage sites.

The peptides varied widely in their sensitivity to proteolysis by NS3/4A. We established a direct relationship between the cleavage efficiency of NS3/4A and the amino acid sequence of its peptide substrates. This dependence is illustrated in the form of sequence logos (Fig 3). The height of a character is proportional to the frequency of the amino acid residue at the individual position of the cleaved peptide.

In agreement with data of others [21]–[27], our results revealed that NS3/4A strongly prefers Cys and Ser at the P1 and P1′ positions, respectively. Clearly, because of the narrow cleavage preferences of NS3/4A and because the peptide substrates were derived from the HCV polyprotein, the C ↓ S pattern was predominant at the P1–P1′ positions. In turn, the promiscuity of amino acid representation was high at the P4–P2 and P2′–P4′ positions of the cleavage peptides. Met, Gln, Gly or Ala were frequently present at the P2′ position of the efficient substrates of NS3/4A. The presence of Cys, Pro and Glu at P2 and Thr, Val and Glu at P3 correlated with increased efficiency of NS3/4A proteolysis. The importance of P3 Val suggested in earlier work [22] appears to have been overestimated because P3 Val was also frequent in the poor substrates of NS3/4A and because both P3 Thr and Glu were frequent in the efficient NS3/4A substrates.

A newly observed and striking feature of NS3/4A was a high frequency of either Asp or Glu at both P5 and P6 positions in a subset of the most efficient NS3/4A substrates. In turn, aberrations of this negative charged sequence such as an insertion of a positively charged or hydrophobic residue between the negatively charged residues resulted in inefficient substrates. Thus, the presence of either Leu, or Arg or Cys at the P5 instead of the negatively charged Glu or Asp was observed in the inefficient peptide substrates of NS3/4A.

### Conventional NS3/4A cleavage sites in the HCV polyprotein

The high number of peptide sequences we tested provided a wealth of information for subsequent analysis. Our data indicate that the peptides that span the known NS3/4A cleavage sites in the HCV polyprotein were efficiently cleaved by the proteinase. Multiple peptides from the NS4A-NS4B junction region (^1705^FDEMEEC↓S^1712^, ^1706^DEMEEC↓SQ^1713^, ^1707^EMEEC↓SQH^1714^, ^1708^MEEC↓SQHL^1715^ and ^1709^EEC↓SQHLP^1716^ with Z-scores of 8.9, 7.0, 6.4, 6.6 and 3.7, respectively), from the NS4B-NS5A junction region (^1965^SSECTTPC^1972^↓, ^1967^ECTTPC↓SG^1974^ and ^1969^TTPC↓SGSW^1976^ with Z-scores of 5.8, 4.3 and 4.3, respectively) and from the NS5A-NS5B region (^2413^NAEDVVCC^2420^↓ ^2415^EDVVCC↓SM^2422^ and ^2417^VVCC↓SMSY^2424^ with Z-scores of 9.3, 91 and 8.9, respectively), were efficiently cleaved by NS3/4A. In contrast, NS3/4A did not cleave any peptides from the NS3-NS4A 1652-1661 junction region: the Z-score of multiple tested peptides was in a 0.8–1.2 range.

The analysis of other, mutant peptides provided supporting data. Thus, we tested multiple mutant peptides the sequences of which were specifically derived from the NS3-NS4A (^1652^DLEVVT↓STWV^1661^), NS4A-NS4B (^1706^DEMEEC↓SQHL^1715^), NS4B-NS5A (^1967^ECTTPC↓SGSW^1976^) and NS5A-NS5B (^2415^EDVVCC↓SMSY^2424^) junction regions. In these mutant peptides, a single position, starting from the N-terminus, was stepwise randomized with 20 amino acids while the other seven positions were fixed. As a result, we generated unique mutant peptides that exhibited 20 amino acids at each of the P6–P4′ positions (Supplemental Table S2). The cleavage analysis of this mutant substrate set supports our conclusion that the NS3-NS4A 1652–1661 junction region is not efficiently cleaved by NS3/4A *in trans*. Table 2 shows a subset our results in the alanine scanning mutagenesis form. The alanine scanning P6–P4′ mutagenesis clearly indicated that multiple mutant peptide sequences derived from the NS4A-NS4B (5 peptides), NS4B-NS5A (5 peptides) and NS5A-NS5B (3 peptides) performed as efficient substrates of NS3/4A. Naturally, the NS4A-NS4B and NS4B-NS5A peptides with the P1 Ala instead of the essential P1 Cys resisted the proteolysis. In contrast, 13 partially overlapping peptides from the NS3-NS4A junction region were resistant to NS3/4A proteolysis. Therefore, in agreement with the results published by others 25,27], we conclude that the cleavage of the NS3-NS4A junction region by NS3/4A takes place *in cis* whereas the NS4A-NS4B, NS4B-NS5A and NS5A-NS5B junctions are readily cleaved *in trans*.

### NS3/4A proteolysis of host cell targets, IPS-1 and TRIF

In agreement with the above conclusions, peptides that represented a potential cleavage site in IPS-1 (^503^EREVPC-HRPS^512^), a potential host cell target of NS3/4A proteolysis [28], were not cleaved in our cleavage tests. It is now clear that an insertion of the positively charged P5 Arg between the Glu/Asp-Glu/Asp negatively charged sequence makes the IPS-1 sequence resistant to NS3/4A proteolysis. In agreement, substitution of the P5 Arg with several amino acid residue types led to the noticeable NS3/4A cleavage of the resulting peptide. In turn, the peptides that span a potential cleavage site of TRIF (^369^STPC-SAHL^376^), another proposed host cell target of NS3/4A proteolysis [29], were readily cleaved by NS3/4A *in vitro* (Supplemental Table S3).

### Potential additional NS3/4A cleavage site in the HCV polyprotein

Our cleavage data suggest that the ^2427^TGALVTPC^2434^↓, ^2429^ALVTPC↓AA^2436^ and ^2431^VTPC↓AAEE^2438^ peptides from the ^2427^TGALVTPC-AAEE^2438^ sequence in the NS5A-NS5B junction region are readily cleaved by NS3/4A *in vitro* (Supplemental Table S1, Tables 1 and 2). The ^2427^TGALVTPC-AAEE^2438^ sequence is 14 residues downstream of the conventional ^2415^EDVVCC-SMSY^2424^ cleavage site in the exposed cytoplasmic loop of the NS5A-NS5B junction (Fig 1). Processing of both cleavage sites of the NS5A-NS5B junction *in vivo* may explain the existence of predominant and minor species of NS5A as observed earlier by Grakoui et al. [18], [30]. The cleavage of the peptides, which were derived from the ^2427^TGALVTPC-AAEE^2438^ region, was observed, however, only following the extended co-incubation with NS3/4A (Supplemental Table S1), thus, suggesting that because of the P5 Leu, P6 Ala and P1′ Ala this peptide sequence is a sub-optimal substrate of NS3/4A.

We also identified several additional peptide sequences, which were efficiently cleaved by NS3/4A *in vitro*. These sequences represented the internal regions of the E1 protein (^269^ATLC-SALY^276^), the E2 protein (^674^VLPC-SFTT^681^), the NS3 helicase domain (^1312^IIIC-DECH^1319^), and the NS5A (^2169^AVLT-SMLT^2176^). These sequence regions, however, are either localized at the opposite side of the membrane relative to NS3/4A (E1 and E2) or in the central region of the NS3 helicase domain and of NS5A and, in contrast with the 2427–2438 sequence of NS5A-NS5B junction, are not readily accessible for NS3/4A proteolysis under physiological conditions *in vivo*.

### Proteolysis of the polyprotein in the HCV quasi-species

Because HCV continually mutates, multiple infective mutant quasi-species of HCV are generated in humans. To corroborate the importance of the NS3/4A cleavage sites we identified in the polyprotein, we analyzed the sequence of the NS3/4A cleavage sites in the known quasi-species of HCV. Our analysis revealed that there are 1895 sequence variants, including EVVTSTWV, EVITSTWV, EVTTSTWV, EVMTSSTWV, EIVTSTWV, EVVTSSWV and EVVTNTWV, of the NS3-NS4A 1654–1661 sequence region of the HCV genotype 1a. In addition, there are 47 sequence variations of the NS3-NS4A cleavage site in the HCV genotype 1b (EVATSTWV, EVVTGTWV, EVVTSAWV, EVVASTWV and EVVTSHWV). Peptides that corresponded to these sequences, however, were not cleaved by NS3/4A *in vitro*. These findings suggest that, exactly as in the HCV genotype 1b we refer to, in all of the known quasispecies of HCV the NS3-NS4A 1654–1661 sequence is also processed by NS3/4A *in cis*.

**Figure 4:**
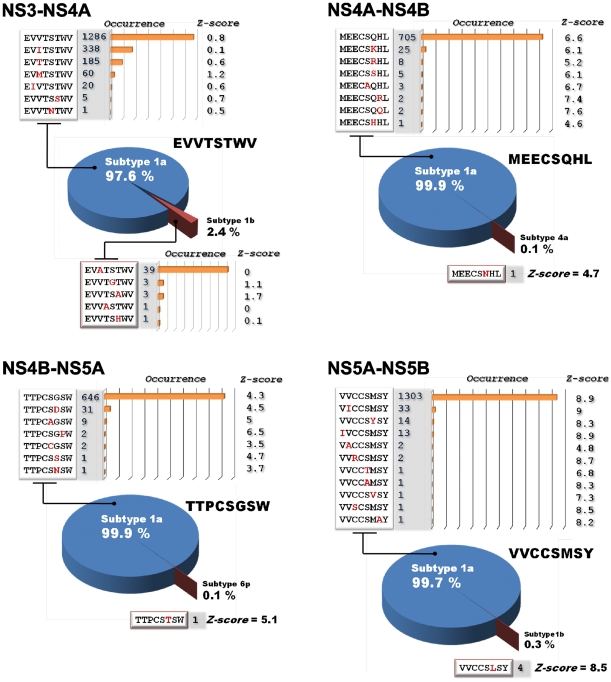
Analysis of the NS3-NS4A, NS4A-NS4B, NS4B-NS5A and NS5A-NS5B junctions in the known HCV isolates. The sequences that correspond to the P4–P4′ positions are shown. Substitutions relative to the HCV genotype 1a sequence (GeneBank Accession P26664) are in red. The frequency of the isolate and the Z-score of the peptide sequences are shown.

In contrast, all of the sequence variants of the NS4A-NS4B 1708–1715 cleavage region (751 variants in the genotype 1a and 1 variant in the genotype 4a) were cleaved as the corresponding peptides in our cleavage tests (Fig 4). All of the peptides that correlated with the sequence of the NS4B-NS5A and NS5A-NS5B junctions in 692 and 1371 HCV quasi-species, respectively, were also cleaved by NS3/4A. We conclude that there are no mutations in the infectious HCV species that make the NS4A-NS4B, NS4B-NS5A and NS5A-NS5B regions resistant to NS3/4A proteolysis. These results support the critical importance of the polyprotein processing by NS3/4A at the NS4A-NS4B, NS4B-NS5A and NS5A-NS5B junction regions *in vivo*.

### Structural evidence for specificity of NS3/4A

**Figure 5:**
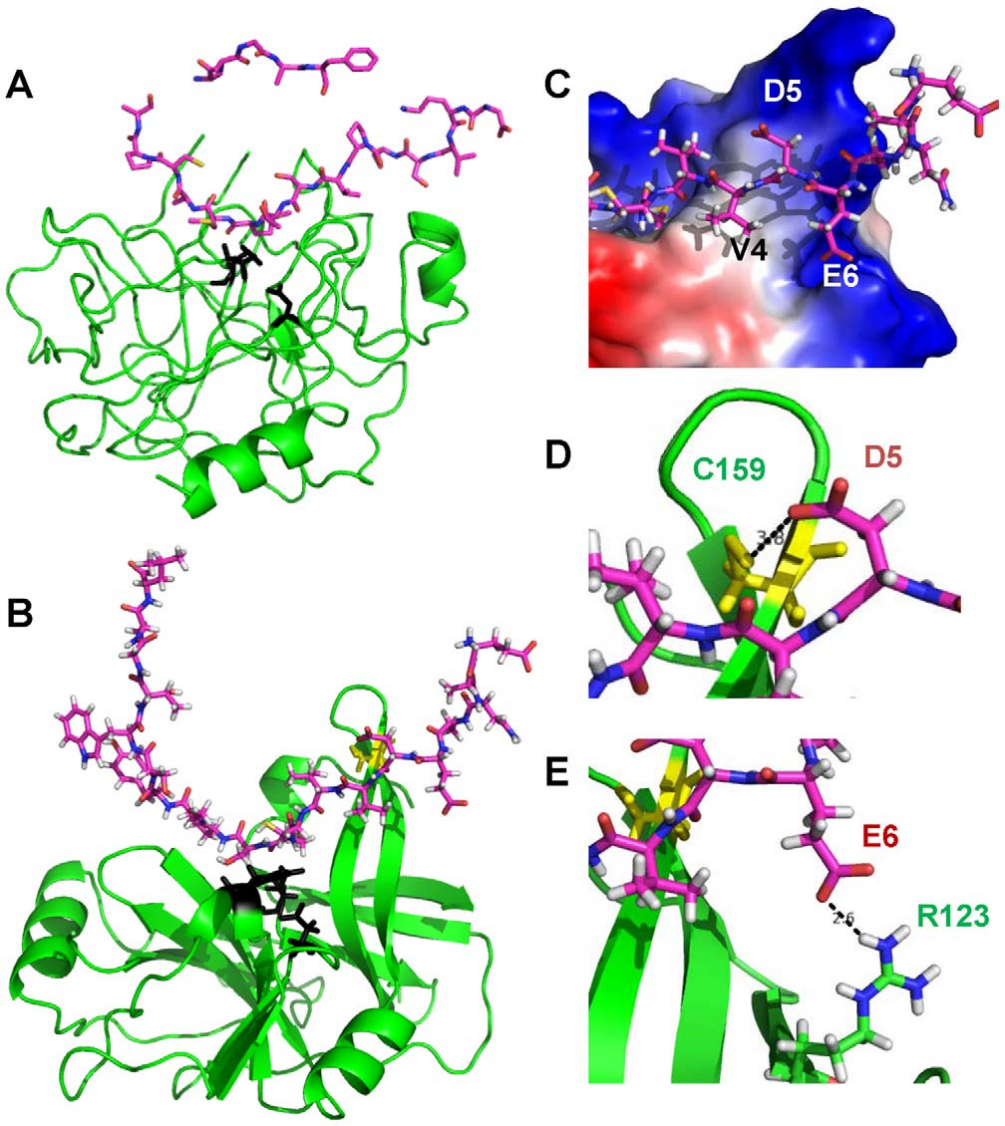
Structural modeling of NS3/4A with a protein substrate. The ^2411^EANAEDVVCC↓SMSYSWTGAL^2430^ peptide that corresponds to the NS5A-NS5B junction region was used as a substrate in our modeling. (A) The structure of chymotrypsin – ecotin complex. This structure (PDB 1N8O) was used as a template for modeling the protein substrate in the NS3/4A structure (PDB 3LOX). Black, the catalytic triad. (B) The structure of the NS3 proteinase domain with the ^2411^EANAEDVVCC↓SMSYSWTGAL^2430^ modeled substrate. Black, the catalytic triad (His-57, Asp-81, Ser-139); yellow, Cys-159. (C) Close-up of the modeled NS3 structure. The negatively charged P5 Asp and P6 Glu of the substrate interact with the positively charged region of the NS3 proteinase that involves Arg-161, Lys-165 and Arg-123 (blue). (D) P5 Asp of the substrate interacts with Cys-159 of the NS3 proteinase. A dashed line shows a 3.8 Å distance between the carboxyl group of P5 Asp and the SH-group of Cys-159. (E) P6 Glu of the substrate interacts with Arg-123 of the NS3 proteinase. A dashed line shows a 2.6 Å distance between the carboxyl group of P6 Glu and the hydrogen of the guanidium group of Arg-123.

To elucidate structural elements that determine the cleavage preferences of NS3/4A, especially at the P5 and P6 positions, we examined the structures of NS3/4A (PDB 3LOX) in its complex with a ketoamide inhibitor derivative of Boceprevir [31] and of chymotrypsin in a complex with its inhibitor, ecotin (PDB 1N8O; Cambillau C., Spinelli S., Lauwereys M., Crystal structure of a complex between bovine chymotrypsin and ecotin at 2.0 Å resolution, to be published) (Fig 5A). Because the overall structure of NS3/4A and chymotrypsin is similar, we used DaliLite software [32] to superimpose 3LOX with 1N8O. Ecotin coordinates served as a template for the binding of a long peptide substrate with NS3/4A. In the 3LOX structure, we modeled the conformation of the peptide substrate that follows main chain atoms of ecotin in 1N8O. Because of Glu and Asp residues are naturally present at the P5 and P6 position of the NS5A-NS5B junction region, we used the latter (^2411^EANAEDVVCC↓SMSYSWTGAL^2430^) in our modeling. The peptide bond between the middle C-S amino acid residues is a scissile bond. We optimized the final position of the substrate using molecular mechanical minimization and limited molecular dynamics simulations using AMBER11 software (33).

Fig 5B shows the modeling results. The P1 and P1′ residues of the substrate were placed in those positions relative to the catalytic triad that are required for the catalysis. The scissile peptide bond was constrained during the molecular mechanical optimization procedure. The inset (Fig 5C) shows that P5 Asp and P6 Glu interact with the positively charged regions of NS3/4A formed by Arg-161, Lys-165 and Arg-123. In addition, our modeling suggests that the carboxyl group of the P5 Asp of the substrate is at a short distance (approximately 3.8 Å) from the SH-group of Cys-159 of the NS3 catalytic domain implying that there could be a strong hydrogen bond between these residues (Fig 5D). Furthermore, the carboxyl group of the P6 Glu side-chain is at a short, 2.6 Å, distance from the hydrogen of the guanidium group of Arg-123 of the NS3 catalytic domain also suggesting a formation of a hydrogen bond (Fig 5E).

## Discussion

HCV is a causative agent of chronic liver disease worldwide with millions of infected patients at risk of morbidity and mortality. The HCV-encoded NS3/4A is essential for viral polyprotein processing and viral replication and has long been considered a promising drug target for pharmacological intervention in HCV-infected patients. In the course of polyprotein processing, NS3/4A cleaves the ^1657^Thr-Ser^1658^, ^1711^Cys-Ser^1712^, ^1972^Cys-Ser^1973^ and ^2420^Cys-Ser^2421^ scissile bonds in the NS3-NS4A, NS4A-NS4B, NS4B-NS5A and NS5A-NS5B junctions, respectively, and generates the essential late viral NS proteins. Based on the cleavage sequence of the junctions, NS3/4A is likely to have a preference for substrates with an acidic residue at P6, Cys at P1 and Ser or Ala at P1′. Multiple substrate specificity studies using synthetic peptides generally confirm this unusually narrow specificity of NS3/4A. These studies resulted in defining a consensus cleavage sequence of NS3/4A as D/E-X-X-X-X-C/T↓S/A-X-X-X, where X is any amino acid residue [22]-[24], [27], [33]. Additional mutagenesis studies, however, have revealed that the P6 residue is dispensable, that the P1′ is tolerant to many residue types (except Pro) and that Cys at the P1 is the dominant determinant for cleavage efficiency [33]. Overall, because of the limited number of synthetic substrates previously employed to characterize NS3/4A, our knowledge of its cleavage preferences is limited as yet. An in-depth knowledge of both cleavage preferences and interactions of NS3/4A with its substrates is required for the structure-based inhibitor design for this HCV proteinase, a prime target of anti-virals.

To fill in this gap in our knowledge, we determined the cleavage preferences of NS3/4A using a novel scalable assay. This assay made use of 8-residue peptide sequences covering the full-length HCV polyprotein. We also analyzed over 800 mutant peptides the sequence of which overlapped those of the NS3-NS4A, NS4A-NS4B, NS4B-NS5A and NS5A-NS5B junctions.

As expected, the C↓S pattern was predominant at the P1–P1′ positions of the efficiently cleaved peptides, supporting the observations by others [18], [23], [27], [33]. Multiple peptides derived from the NS4A-NS4B, NS4B-NS5A and NS5A-NS5B junction regions were efficiently cleaved by NS3/4A. Selective cleavage of these peptide sequences corroborate the known NS3/4A cleavage sites in the HCV polyprotein and, in addition, validate both the precision and selectivity of our multiplex cleavage assay technology. In turn, the peptides derived from the NS3-NS4A 1654-1661 junction region were highly resistant to NS3/4A proteolysis. The resistance of the NS3-NS4A junction to the *in trans* cleavage by NS3/4A are consistent with the results by others [3], [27] and suggests that the cleavage of this site *in vivo* takes place *in cis*. Alternatively, the *in vivo* cleavage may require either structural re-arrangements, which are not understood as yet, or additional co-factors or both [34].

A new observation was that NS3/4A strongly prefers Asp and Glu at the P5 and P6 positions in a subset of the efficient cleavage substrates. Our data suggest that the presence of this negatively charged residue pair at these substrate positions is a strict requirement for the most efficient substrate cleavage by NS3/4A *in vitro*. This feature explains the efficiency of NS3/4A in cleaving the NS4A-NS4B, NS4B-NS5A and NS5A-NS5A junctions *in vivo* each of which exhibits the negative charged pair at either the P6–P5 or P7–P6 positions in multiple HCV quasispecies.

Our *in vitro* data imply that there could be an additional site (^2431^VTPC↓AAEE^2438^) in the HCV polyprotein for NS3/4A cleavages *in vivo*. This putative site is downstream of the conventional ^2417^VVCC↓SMSY^2424^ cleavage site in the NS5A-NS5B junction. It is tempting to hypothesize that ^2427^TGALVTPC-AAEE^2438^ sequence comprises an additional NS3/4A cleavage site in the HCV polyprotein. A possible physiological significance of the putative ^2431^VTPC↓AAEE^2438^ cleavage site is to release of the NS5B RNA polymerase, an essential component of the HCV replicase complex.

HCV continually mutates while it replicates. As a result, thousands of infective mutant quasi-species of HCV have already been identified. There are multiple HCV isolates with mutations in the NS3/4A cleavage site sequences. Our analysis, however, revealed that there are no mutations that inactivate the NS3/4A cleavage sites in over 5000 known HCV isolates. These results indicate the critical importance of the polyprotein processing by NS3/4A at the NS4A-NS4B, NS4B-NS5A and NS5A-NS5B junction regions *in vivo*. Conversely, the NS3-NS4A junction sequences from over 1500 isolates were resistant to NS3/4A proteolysis *in vitro*, suggesting that there are no variants of HCV in which the NS3-NS4A junction is cleaved by NS3/4A *in trans*.

To elucidate structural requirements, which determine the preference of NS3/4A for a Glu-Asp pair at the P5 and P6 positions, we modeled NS3/4A in its complex with the peptide substrate. For this purpose, we used the atomic resolution structure of NS3/4A (PDB 3LOX) 31] and of the chymotrypsin-ecotin complex (PDB 1N8O; Cambillau C., Spinelli S., Lauwereys M., to be published). In the latter, ecotin, a tight-binding protein inhibitor, acts as a substrate mimic. Ecotin coordinates from PDB 1N8O were used as a template for modeling of our 20 amino acid residue long peptide substrate which represented the NS5A-NS5B junction region of the HCV polyprotein. As a result of this modeling, we now understand that the requirements for Cys at the P1 and Ser at the P1′ insufficiently describe the cleavage preferences of NS3/4A and that long-range interactions with the substrate are also critical for NS3/4A. Thus, it is likely that Asp and/or Glu at the P5 and P6 positions interact with a positively charged region in the NS3/4A molecule. This region is formed by Arg-161, Lys-165 and Arg-123 of NS3/4A.

Our modeling also suggests that the carboxyl group of the Asp P5 is at a short distance (3.8 Å) from the SH-group of Cys-159, implying the existence of a strong hydrogen bond between the Asp P5 and the functionally important non-catalytic Cys-159 residue 35]. Furthermore, the carboxyl group of the Glu P6 side-chain is proximal to the hydrogen of the guanidium group of Arg-123 (2.6 Å) also suggesting formation of a hydrogen bond. It becomes clear that the requirement for P5 and P6 Glu and Asp is embedded in the NS3/4A structure. In turn, regardless the presence of P1 Cys and P1′ Ser in their sequence, peptides with aberrations in the Asp-Glu P5–P6 tandem are inefficiently cleaved by NS3/4A. Overall, our findings suggest the long-range interactions with the substrate plays a significant role in the NS3/4A functionality and that the (E/D)(E/D)XXXC ↓ S motif represents the global signature of the NS3/4A cleavage preferences.

## Materials and Methods

### Reagents

Reagents were purchased from Sigma-Aldrich unless indicated otherwise. NS3/4A was purchased from AnaSpec (Fremont, CA). Furin and the two-component NS2B-NS3 proteinase from Dengue and West Nile viruses were isolated and characterized as described earlier [36]–[38].

### Peptide synthesis and cleavage assay

Peptide synthesis and both the precise methodology of the *in vitro* cleavage assay and of measuring and registering the peptide cleavage levels will be published elsewhere (Kozlov IA, Thomsen ER, Munchel SE, Villegas P, Capek P^,^ Gower AJ, Pond P, Chudin E and Chee MS. A Highly Scalable Peptide-Based Assay System for Proteomics, submitted). Briefly, *in vitro* transcription was used to convert a pool of DNA templates (prepared by a microarray-based synthesis) into a pool of RNAs. *In vitro* translation was then used to generate a pool of peptides covalently linked *via* their C-terminus to their RNA templates [39]. To increase their stability, the peptide-RNA fusions were converted to the corresponding covalent peptide-cDNA fusions [40]. The peptide-cDNA fusions were immobilized on magnetic beads using an affinity tag attached to the N-terminus of all peptides. As a result of a proteolytic cleavage of the immobilized peptide-DNA fusions, the corresponding cDNA templates were released from the beads into solution. Peptide substrates cleaved by a proteinase were identified *via* high throughput sequencing of the released cDNAs using a Genome Analyzer IIx (Illumina, San Diego, CA).

In our current study, we produced an over 2,660 peptide set that contained two groups of 8-mer peptide sequences. The first group consisted of 1,503 overlapping peptide sequences that, when combined, covered the full-length sequence of the HCV polyprotein. There was a two residue offset resulting in a 6 residue overlap of the upstream and downstream peptides. The second group consisted of over 1,000 mutant peptides which represented variations of the NS3-NS4A, NS4A-NS4B, NS4B-NS5A and NS5A-NS5B junctions. To monitor the assay performance, this set also included positive and negative controls (68 peptides). Positive controls included the known cleavage sequences of thrombin, furin, enterokinase, the NS2B-NS3 proteinase from West Nile virus, and several additional commercially available proteinases. Negative controls included deca-Gly, deca-Ala, (Gly-Ala)x5, (Ala-Gly)x5 peptides, and no peptide. The methods described in the current publication were also validated using other proteinases including furin, thrombin, enterokinase, and NS2B-NS3 and NS3/4A proteinases from West Nile virus and HCV, respectively. According to our cleavage results, there was no overlap among the specific peptide sets each of which was predominantly cleaved by the expected proteinase alone.

### Proteinase assay

The cDNA-peptides fusions were immobilized on magnetic beads via the N-terminus and treated with HCV NS3/4A proteinase. Reactions without proteinase added were used as negative controls (no proteinase controls). DNA molecules released by peptide cleavage were collected from each sample and sequenced following the attachment of adapter sequences by PCR [41].

### Cleavage data analysis

Peptide abundance in solution was quantified by counts of DNA reads corresponding to each peptide sequence. The cleavage levels were estimated by comparing the log-transformed counts in the proteinase-treated *versus* the untreated samples. We used a locally weighted scatter plot smoothing fit as implemented in the lowess (locally weighted scatterplot smoothing) function from the statistical analysis package R to adjust for sequence-specific variance in abundance levels. The residuals of the fit were modeled as arising from a mixture of two distributions with different means. The main peak with mean of residuals equal to 0 (due to lowess robustness) corresponded to the intact peptides and the second peak with positive mean corresponded to cleaved peptides. The robust standard deviation of residuals was computed using the median absolute deviation estimator after which residuals were converted to Z-scores. After this transformation, Z-scores of intect peptides were assumed to be distributed as a standard normal variable. Statistical significance was inferred by converting Z-scores to p-values and adjusting for multiple hypotheses testing using false discovery rate (FDR) [42]. We chose to reject the hypothesis of no cleavage at Z-score >3, which corresponds to nominal p<0.0014 and FDR<0.01. When in question, the identity of the scissile bonds in certain peptides was confirmed in the cleavage experiments followed by mass-spectrometry analysis of the digest.

The sequence logos were obtained by calculating cleavage efficiency for NS3/4A over the entire set of substrates and then selecting the substrates with the cleavage efficiency equal or above the Z-score  = 3 threshold. These substrates were considered susceptible to NS3/4A proteolysis. In turn, the substrates with the cleavage efficiency below threshold form a separate group which was considered resistant to NS3/4A proteolysis. The resulting logos were created by a web-based IceLogo program [43].

### Sequence analysis of HCV quasi-species

The HCV peptides, containing identified cleavage sites, were aligned against known HCV genomes using BLAST [44]. The alignment data were processed using Biopython [45], which is a collection of tools for computational biology and bioinformatics, written in the Python scripting language. Quasi-species with multiple substitutions in the regions of interest were excluded from the analysis.

### Modeling

Molecular mechanical calculations were performed using the Amber11 molecular modeling package [46] and ff99SB force field [47]. We applied the Generalized Born method [48] to represent solvent as a continuous medium in all calculations. Optimization of the built NS3 proteinase-substrate complex involved consecutive short molecular dynamics simulations followed by the energy minimization of the substrate. In the course of the molecular dynamics and minimization steps, the orientations of the backbone heavy atoms of the P1 and P1′ residues were kept constrained to the positions that are required for proteolytic cleavage to occur.

## Supporting Information

Table S1 **The sequence and cleavage efficiency of the 8-residue peptides we synthesized and tested in the cleavage reactions with NS3/4A.** The cleavage efficiency of these peptides by NS3/4A was measured in the 7.5, 15 and 240 min cleavage reactions. The peptides with Z-scores above 3 are shaded pink.
(XLSX)

Table S2 **NS3/4A proteolysis of the mutant peptides derived from the NS3-NS4A, NS4A-NS4B, NS4B-NS5A and NS5A-NS5B junction regions.** The sequence of the 8-residue peptides was specifically derived from the NS3-NS4A (^1652^DLEVVT↓STWV^1661^), NS4A-NS4B (^1706^DEMEEC↓SQHL^1715^), NS4B-NS5A (^1967^ECTTPC↓SGSW^1976^) and NS5A-NS5B (^2415^EDVVCC↓SMSY^2424^) junction regions. A single position, starting from the N-terminus, was stepwise randomized with 20 amino acids while the other seven positions were fixed. As a result, a set of mutant peptides that exhibited 20 amino acids at each of the P6-P4′ positions was synthesized. The cleavage efficiency of these peptides by NS3/4A was measured in the 7.5,15 and 240 min cleavage reactions. The peptides with Z-scores above 3 are shaded pink. The wild-type sequences are grey. Yellow color indicates a few peptides that failed our internal quality control indicating a problem with synthesis.
(XLSX)

Table S3 **NS3/4A proteolysis of the mutant peptides derived from the sequence of TRIF and IPS-1.** The sequence of the 8-residue peptides was specifically derived from the potential NS3/4A cleavage sites in TRIF and IPS-1 (^369^STPC↓SAHL^376^ and ^503^EREVPC↓HRPS^512^, respectively). A single position, starting from the N-terminus (except the P1 and P1′ positions), was stepwise randomized with 20 amino acids while the other seven positions were fixed. As a result, a set of mutant peptides that exhibited 20 amino acids at each of the P6–P4′ positions was synthesized. The cleavage efficiency of these peptides by NS3/4A was measured in the 7.5,15 and 240 min cleavage reactions. The peptides with Z-scores equal or above 3 are shaded pink. The wild-type sequences are grey.
(XLSX)
